# Cold Tolerance and Differential Expression of Cuticular Protein Genes in *Sungaya inexpectata* Zompro, 1996 (Insecta: Phasmatodea)

**DOI:** 10.3390/insects17060604

**Published:** 2026-06-08

**Authors:** Kun Yang, Xuxiang Wu, Yihan Chen, Wenjing Ma, Yijie Lin, Xingzhou Ma, Jiayong Zhang

**Affiliations:** 1 College of Life Sciences, Zhejiang Normal University, Jinhua 321004, China; 2 MOE Frontier Science Center for Brain Science and Brain-Machine Integration, State Key Laboratory of Brain-Machine Intelligence, Zhejiang University, Hangzhou 311121, China; 3 Key Lab of Wildlife Biotechnology, Conservation and Utilization of Zhejiang Province, Zhejiang Normal University, Jinhua 321004, China

**Keywords:** *Sungaya inexpectata*, cold stress, cuticular protein family, qRT-PCR, transcriptome

## Abstract

*Sungaya inexpectata* is a tropical stick insect endemic to the Philippines, providing a useful system for investigating cold responses in tropical ectotherms. In this study, we exposed individuals to low temperature (8 °C) and normal temperature (25 °C) and characterized their transcriptomic responses. A total of 1656 differentially expressed genes were identified, including those involved in energy metabolism, cuticular proteins (CPs), and heat shock proteins. Since CP-related genes were notably enriched, we focused on this family. qPCR assessment provided preliminary expression profiles for selected candidate CP genes. Using comparative transcriptomics with eight New Zealand alpine stick insect species, we reconstructed the phylogeny of major CP families and annotated their conserved domains. Clade analysis revealed significant positive selection in the *CPAP3-3* gene. In summary, this study reveals the transcriptional response of cuticular protein genes in *S. inexpectata* under cold exposure at 8 °C. These findings provide preliminary transcriptional data for understanding how this species responds to low temperature.

## Simple Summary

An increasing number of tropical stick insects are being kept as pets in China, providing an opportunity to investigate how tropical ectotherms respond to low-temperature stress. However, current knowledge of their cold tolerance remains limited. This study investigates the mechanisms underlying the low-temperature response of the tropical stick insect *Sungaya inexpectata*, which has been increasingly observed in temperate areas. Transcriptomic profiling of cold-exposed individuals revealed upregulation of multiple genes. Among the top 20 significantly differentially expressed genes, several genes encoding cuticular proteins were identified, prompting us to further investigate these genes. We identified one cuticular protein gene showing a significant positive selection signal, suggesting that this gene may be a candidate for future studies of cuticle-related responses to cooler environments. These findings provide preliminary data on the transcriptional response of *S. inexpectata* to low temperature.

## 1. Introduction

Under climate warming, numerous species are undergoing poleward and elevational range shifts, which may expand their geographical distributions and thereby reshape local ecological assemblages [1]. Concurrently, an increasing number of tropical stick insect species are being imported into China for the pet trade and subsequently maintained in private collections. During laboratory rearing, we observed that *Sungaya inexpectata*, a tropical stick insect endemic to the Philippines, can overwinter in indoor enclosures under controlled low-temperature conditions. Additionally, it exhibits broad folivory, feeding on leaves of multiple plant species including guava (*Psidium guajava*), rose (*Rosa* spp.), honeysuckle (*Lonicera japonica*), and mulberry (*Morus alba*), and possesses obligate or facultative parthenogenesis [2]. Notably, documented outbreaks of other stick insect species, such as a case in Japan, have caused severe defoliation and forest regeneration failure [3]. These observations raise the possibility that if accidentally or intentionally released into local ecosystems, this species might establish populations and potentially cause ecological impacts.

Temperature is one of the most decisive abiotic drivers shaping insect distribution, phenology, and life-history strategies [4]. Exposure to extreme temperatures can impair developmental stability, diminish key fitness components (e.g., juvenile survival rate, adult longevity, fecundity, and dispersal ability), and ultimately constrain population viability and geographic persistence [5,6,7,8]. Although *S. inexpectata* is intrinsically adapted to tropical thermal regimes, studies on other insects have demonstrated that realized geographic distributions are strongly delimited by low-temperature thresholds [9], particularly winter minima and cumulative chilling degree days, suggesting that low-temperature tolerance may also influence the establishment potential of *S. inexpectata*. Collectively, the physiological resilience traits and ecological generalism of *S. inexpectata* position it as a useful non-model system for investigating molecular responses to low-temperature stress.

**Figure 1:**
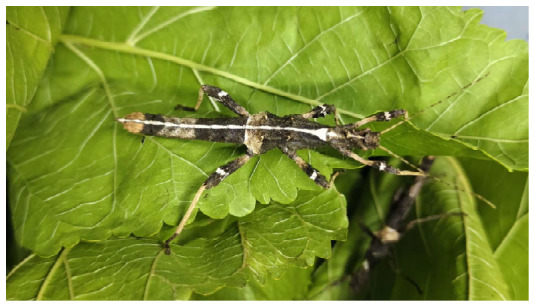
*Sungaya inexpectata* photographed in the laboratory.

*Sungaya inexpectata* (Phasmatodea: Phasmatidae) is a brown-and-white striped stick insect endemic to the tropical Philippines (Figure 1) [10]. Comparative phylogeographic and physiological studies of New Zealand phasmids reveal that alpine lineages have convergently evolved two functionally distinct cold tolerance strategies: chill tolerance and freeze avoidance, each tightly coupled to the prevailing thermal characteristics of their respective microhabitats. Notably, cuticular protein genes in these alpine stick insects exhibit statistically significant signals of positive selection, suggesting that they may contribute to survival in low-temperature and hypoxic environments [11,12,13]. These findings provide a useful comparative framework for investigating cold-responsive genes, particularly cuticular protein genes, in *S. inexpectata*.

In recent years, stick insect research has expanded rapidly across areas such as thermal physiology under extreme temperatures and integrative phylogenomics [11,13,14]. At the same time, advances in high-throughput transcriptomics, especially RNA-Seq–based gene expression profiling, have enabled systematic identification of temperature-responsive pathways and candidate effector genes across diverse insect taxa [15,16,17]. Comparative RNA-Seq analyses of New Zealand alpine stick insects have shown that cold stress triggers extensive transcriptional reprogramming, involving genes related to transcriptional regulation, central carbon metabolism, mitochondrial energy transduction, and cuticular organization. Among these, cuticular protein (CP) genes showed particularly strong and consistent transcriptional responses [13]. As major structural components of the insect exoskeleton, CPs are essential for cuticle biosynthesis, biomechanical stability, desiccation tolerance, and the maintenance of the interface between the insect body and the external environment [18]. Genomic and transcriptomic evidence indicates that insect CPs comprise multiple evolutionarily conserved subfamilies [15], with substantial interspecific variation in gene copy number and sequence divergence. However, empirical data on how CP genes respond to low-temperature stress in *S. inexpectata* remain lacking.

From a macroevolutionary perspective, interspecific variation in cold tolerance is pronounced, and low-temperature stress can act as an important selective pressure shaping these differences. Because the cuticle functions as a major physical barrier between insects and their external environment, changes in cuticular structure and composition may contribute to cold tolerance. The cuticular protein family exhibits substantial molecular diversity and lineage-specific variation [19,20,21]. Previous studies have suggested that CP genes may respond to thermally variable habitats through changes in expression, sequence evolution, and physicochemical properties [13,22,23,24]. For example, cuticular structure-related genes in New Zealand alpine stick insects show both differential expression under cold exposure and signals of positive selection [13]. Based on these findings, we hypothesized that CP genes in *S. inexpectata* may be transcriptionally responsive to low-temperature stress and may show evolutionary signatures associated with cold adaptation.

In this study, we used RNA-Seq and de novo transcriptome assembly to generate a transcriptome resource for *S. inexpectata* and to characterize transcriptional responses elicited by acute cold exposure. Differential expression analysis identified candidate genes responsive to low-temperature stress, including multiple genes encoding cuticular proteins. To place these findings in an evolutionary context, we integrated transcriptomic and coding sequence data from eight New Zealand alpine stick insect species together with *S. inexpectata*, allowing phylogenetic reconstruction of major cuticular protein families. We further performed positive selection analyses using clade model C (CmC), with the branch leading to *S. inexpectata* designated as the foreground branch. Finally, we examined the expression profiles of selected CP-encoding genes using qRT-PCR as a preliminary expression assessment. Overall, this study provides a transcriptomic resource for understanding molecular responses to low-temperature stress in this tropical stick insect and identifies candidate cuticular protein genes for future functional investigation.

## 2. Materials and Methods

### 2.1. Insect Rearing and Experimental Treatment

*S. inexpectata* is a stick insect native to the tropical Philippines and has been introduced into private collections through the pet trade. Insects were obtained from a commercial pet store, and colonies were reared via parthenogenesis under laboratory conditions. Nymphs were fed fresh guava leaves. Rearing was conducted at 25 °C under a 12 h light:12 h dark photoperiod with 60–70% relative humidity.

Based on published protocols [25] and our preliminary experiments, 8 °C was selected as the cold stress temperature for the main experiment. At the onset of the second instar, six individuals were selected and randomly assigned to two groups: a cold-exposed group (8 °C for 24 h) and a control group (25 °C for 24 h), with three biological replicates per group. Immediately after cold exposure or control incubation, all individuals were snap-frozen in liquid nitrogen and stored at −80 °C until RNA extraction for downstream transcriptome profiling and qRT-PCR expression assessment.

### 2.2. Transcriptome Sequencing, Assembly, and Principal Component Analysis

Total RNA was extracted from whole nymph bodies using TRIzol reagent (Takara, Dalian, China) according to the manufacturer’s protocol. RNA concentration and purity were assessed using a NanoDrop 2000 spectrophotometer (Thermo Fisher Scientific, Waltham, MA, USA). High-quality RNA samples were submitted to Berry Genomics Corporation (Beijing, China) for stranded, poly(A)-selected RNA sequencing library construction. Poly(A)-enriched mRNA was isolated using Oligo(dT) magnetic beads and chemically fragmented to an average length of ~300 bp. Using these fragments as templates, first-strand cDNA was synthesized with random hexamer primers and reverse transcriptase, followed by second-strand synthesis to generate double-stranded cDNA. After library construction, fragments were enriched by PCR amplification, and libraries of approximately 450 bp were selected. Library quality and concentration were assessed using an Agilent 2100 Bioanalyzer (Agilent Technologies, Santa Clara, CA, USA). Paired-end sequencing (2 × 150 bp) was then performed on the Illumina NovaSeq 6000 platform (Illumina, San Diego, CA, USA), yielding at least 6 Gb of raw data per sample.

Raw sequencing reads were subjected to rigorous quality control and adapter trimming using fastp v0.24.3 [26]: bases with quality scores below Q20 were trimmed from read ends, Illumina universal adapters were removed, and reads shorter than 50 nt after trimming were discarded, resulting in high-quality clean reads. De novo transcriptome assembly was performed on the clean reads using Trinity v2.14.0 [27] with parameters appropriate for strand-specific RNA-seq libraries. To reduce redundancy, transcripts were clustered using CD-HIT v4.8.1 [28] with a sequence identity threshold of 95%, and the longest representative transcript from each cluster was retained. Open reading frames (ORFs) were predicted from the resulting non-redundant transcript set using TransDecoder v5.7.1 [29]. Assembly completeness was assessed via BUSCO analysis against five orthologous databases: bacteria_odb10, eukaryota_odb10, insecta_odb10, metazoa_odb10, and viridiplantae_odb10.

The high-quality clean reads were aligned to the non-redundant transcript set to generate alignment files for subsequent expression quantification. Gene-level expression abundance was estimated using RSEM v1.3.3 [30] with default parameters. The raw count matrix was filtered to remove genes with low expression. The filtered count matrix contained three biological replicates of the 8 °C cold-exposed group and three replicates of the 25 °C control group. Principal component analysis (PCA) was performed using log2-transformed TPM values to visualize sample relationships. The results were displayed as a scatter plot, and 95% confidence ellipses were added for the two groups.

### 2.3. Differential Expression Analysis, GO Annotation, and Enrichment Analysis

Differential expression analysis between cold-exposed (8 °C) and control (25 °C) groups was conducted using DESeq2 v1.34 [31] with thresholds of |log_2_FoldChange| ≥ 2 and FDR < 0.05. Differentially expressed genes (DEGs) were classified as significantly upregulated (log_2_FoldChange ≥ 2, FDR < 0.05) or downregulated (log_2_FoldChange ≤ −2, FDR < 0.05). To visualize the expression patterns of DEGs, a volcano plot was generated using ggplot2 [32], with threshold lines indicating the DEG cutoffs.

Functional annotation of the assembled transcripts was performed using the Trinotate [29] pipeline. Using DIAMOND [33] for rapid sequence alignment against the SwissProt database and HMMER [34] for Pfam [35] domain searches, the pipeline generated a TSV output file containing gene and transcript IDs, SwissProt top hits, Pfam domains, and GO terms [36].

To prepare for functional enrichment analysis, gene-associated GO terms were extracted from the annotation file. A Python v3.12.0 script was then used to integrate the GO annotation with the gene expression matrix, creating a structured annotation database linking each gene to its GO labels. Significantly up- and down-regulated DEGs were subjected to GO enrichment analysis using TBtools v1.098 [37]. GO terms with adjusted *p*-values < 0.05 were considered significantly enriched. For visualization, the top 10 enriched terms with the smallest adjusted *p*-values in each GO category were selected. The filtered results were then visualized in RStudio v4.5.0.

### 2.4. Comparative Transcriptomics and Domain Identification

Raw RNA-Seq data from eight cold-exposed New Zealand alpine stick insect species [14] were downloaded from the NCBI Sequence Read Archive (SRA) https://www.ncbi.nlm.nih.gov/sra/ (accessed on 25 November 2025) (*Asteliaphasma jucundum*, *Tectarchus salebrossus*, *Niveaphasma annulata*, *Tectarchus ovobesus*, *Argosarchus horridus*, *Acanthoxyla* sp., *Clitarchus hookeri*, *Spinotectarchus acornutus*). To ensure analytical consistency, these datasets underwent identical preprocessing as the *S. inexpectata* transcriptome: raw reads were subjected to stringent quality control and adapter trimming using fastp v0.24.3 [26]; de novo transcriptome assembly was performed via Trinity v2.14.0 [29] under strand-specific parameters, yielding transcript assemblies for each of the eight species. After integrating the *S. inexpectata* transcript FASTA set, a transcript library covering all nine stick insect species was constructed. To reduce biases from de novo transcriptome assembly (e.g., isoform collapse and fragmentation), the longest representative transcript from each cluster was retained for orthology inference.

Subsequently, open reading frames (ORFs) and coding sequences (CDSs) were identified from the assembled transcripts of each species, and the corresponding amino acid sequences were obtained by translation. These protein sequences were used as input for OrthoFinder [38] to identify single-copy orthologous genes among the nine stick insect species. A Python script was used to identify orthogroups shared between *S. inexpectata* and at least one of the other stick insect species. For these orthogroups, domain queries were performed using the InterPro [39] website https://www.ebi.ac.uk/interpro/ (accessed on 13 December 2025) to obtain functional annotations.

The predicted protein sequences were searched against six cuticular protein HMM profiles (CPR, CPAP, CPF, TWDL, CPCFC, CPLCA) downloaded from the InterPro [39] website https://www.ebi.ac.uk/interpro/ (accessed on 13 December 2025) to identify candidate cuticular protein sequences. For each protein family, hmmsearch was used to obtain candidate sequences. Candidate cuticular proteins containing the Rebers and Riddiford Consensus (CPR) family were further classified into RR1 and RR2 subfamilies via the CutProtFam-Pred web server [40] http://aias.biol.uoa.gr/CutProtFam-Pred/ (accessed on 10 January 2026) using an E-value threshold of <5.0 × 10^−7^ [41], CPAP subfamily proteins were divided into CPAP1 and CPAP3 subfamilies using default scores.

After obtaining classified sequences, multiple sequence alignment was performed using MAFFT [42]. The aligned sequences were further processed with Gblocks [43] to remove poorly aligned and highly variable regions, retaining reliable conserved regions to reduce phylogenetic noise. Phylogenetic trees were then constructed with IQ-TREE2 v2.3.6 [44]. For families with too few sequences (e.g., CPF, TWDL, CPCFC, CPLCA), tree building was not performed. Subsequent visualization and optimization of the resulting trees (CPR1, CPR2, CPAP1, CPAP3) were conducted using the online tool iTOL [45] (https://itol.embl.de) (accessed on 4 June 2026), where branches with bootstrap support values between 70 and 100 were annotated. Conserved protein motifs were identified using the MEME web server https://meme-suite.org/meme/tools/meme (accessed on 25 January 2026).

### 2.5. Phylogenetic Analyses and Selection Pressure Analysis

Single-copy orthologous genes shared by all nine stick insect species, including *S. inexpectata*, were used for phylogenetic reconstruction. The original nucleotide sequences of these genes were extracted from assembled transcript files. All target sequences were extracted using PhyloSuite v1.2.3 [46] and aligned at the codon level using MAFFT v7.475 [42]. Poorly aligned and highly variable regions were removed using Gblocks v0.91b [43]. The filtered sequences were concatenated using the built-in module of PhyloSuite. IQ-TREE2 [44] was used to construct a phylogenetic tree.

Positive selection analyses were conducted using EasyCodeML v1.07 [47]. The Clade model C (CmC) was used as the alternative model, allowing the foreground branch (leading to *S. inexpectata*) to have an independent *ω* value. The M2a_rel model, which constrains the foreground and background branches to share the same *ω* ratio (*ω*_2_ = *ω*_3_) [48], was used as the null model. Significance was assessed using the likelihood ratio test (LRT), followed by Benjamini–Hochberg false discovery rate correction for multiple testing. Genes with FDR-adjusted q-values < 0.05 were considered statistically significant. The branch leading to *S. inexpectata* was labeled as the foreground branch.

### 2.6. qRT-PCR Expression Assessment

For qRT-PCR expression assessment, four individuals of *S. inexpectata* were randomly selected from the control (25 °C) and low-temperature (8 °C) groups. The insects were sacrificed and placed into 1.5 mL RNase-free microcentrifuge tubes to ensure RNA integrity for subsequent molecular analysis. Tissue samples were snap-frozen in liquid nitrogen and subsequently stored at −80 °C. Total RNA was extracted from all eight samples using the Animal Tissue Total RNA Extraction Kit (Forgene Company, Chengdu, China). RNA integrity was assessed by visualizing distinct 28S/18S rRNA bands on agarose gels. To eliminate genomic DNA contamination, RNA was treated with DNase at 42 °C for 2 min using the PrimeScript™ RT Reagent Kit (Takara Bio Inc., Kusatsu, Shiga, Japan). cDNA synthesis was then performed with the same kit under the following conditions: 37 °C for 15 min (reverse transcription), 85 °C for 5 s (enzyme inactivation), and a final hold at 4 °C.

A heatmap of cuticular protein gene expression was generated using TBtools [37]. Six significantly differentially expressed cuticular protein genes of *S. inexpectata* were selected for qRT-PCR expression assessment. The *β-actin* gene [49,50] was used as the reference gene. Gene-specific primers for the six target genes and the actin gene were designed using Primer Premier 6.0 software [51]. Primer sequences are provided in Table 1.

Using the EASY Dilution method, cDNA from each sample was serially diluted to five concentration gradients: 10^−1^, 10^−2^, 10^−3^, 10^−4^, and 10^−5^. These dilutions were used to generate standard curves and to check whether the amplification efficiencies of the primers were similar. Three technical replicates were performed for each primer pair to ensure accuracy. Transcript levels were quantified using the StepOnePlus™ Real-Time PCR System (Life Technologies, Carlsbad, CA, USA). All reaction components except the cDNA template were first prepared in microcentrifuge tubes, vortexed, and mixed thoroughly. The thermal cycling protocol included an initial denaturation at 95 °C for 30 s, followed by 40 cycles of denaturation (95 °C, 5 s) and annealing/extension (55 °C, 30 s).

Transcript levels of the six selected cuticular protein genes were determined using cycle threshold (Ct) values. The Ct value is defined as the number of amplification cycles needed for the fluorescent signal to reach the detection threshold. Relative gene expression was calculated using the 2^(^−^ΔΔCt) method. In this method, ΔCt was obtained by subtracting the Ct value of β-actin from that of the target gene. Data from four biological replicates were presented as mean ± standard error (SE). Intergroup differences were assessed using independent-sample *t*-tests performed with SPSS v21.0 [52] (SPSS, Inc., Chicago, IL, USA).

## 3. Results

### 3.1. Results of Transcriptome Sequencing, Assembly, and Principal Component Analysis

To characterize the transcriptomic response of *S. inexpectata* to cold stress, a total of 120.64 Gb of high-quality, adapter-trimmed clean data were obtained. Owing to poor RNA quality in some samples that failed library construction and sequencing, six samples (B254–B256 and B84–B86) were retained for subsequent transcriptome analysis. Six cDNA libraries were generated, comprising three cold-exposed individuals at 8 °C and three controls at 25 °C, with a mean Q30 base percentage above 96% across all samples (Table S1).

The final assembly comprised 414,062 transcripts derived from 302,864 putative genes, with a cumulative length of 548 Mb and a GC content of 40.24%. The assembly N50 length was 3383 bp across all transcripts and 1620 bp for the longest isoform per locus (Table S2). The transcript length distribution included both long and short transcripts, reflecting the inherent heterogeneity of de novo transcript reconstruction from short-read RNA-seq data. BUSCO analysis against the insecta_odb10 database revealed that 89.10% of the 1367 insect universal single-copy orthologs were recovered as complete. BUSCO analyses against the eukaryota_odb10 and metazoa_odb10 databases yielded completeness values of 88.5% and 87.9%, respectively. The low recovery of bacterial BUSCO genes (bacteria_odb10, 12.4% complete) suggested limited bacterial contamination in the assembly (Figure S1). These results indicated that this assembly was sufficiently complete for downstream functional annotation and differential expression analysis.

**Figure 2:**
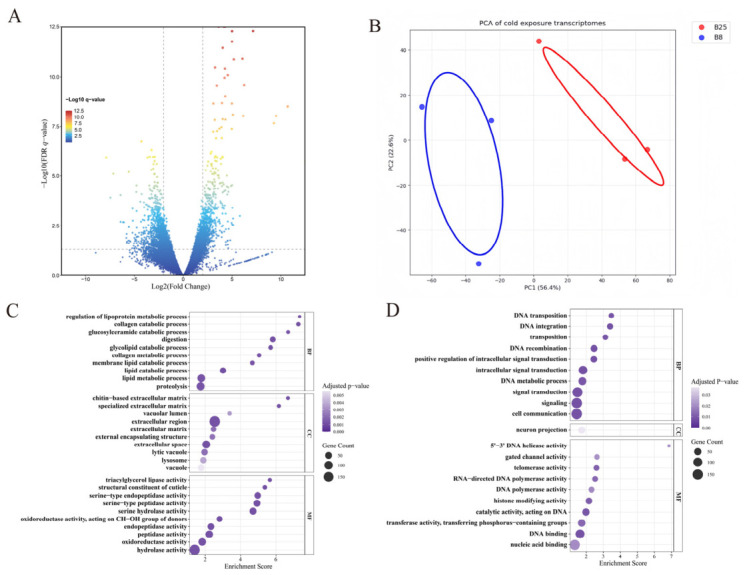
Volcano plot, GO enrichment plots, and PCA plot of transcriptome data from *S. inexpectata* under cold stress. (**A**) Volcano plot (|log_2_FoldChange| ≥ 2, FDR < 0.05). (**B**) Principal component analysis (PCA) plot. (**C**) GO enrichment of cold-upregulated genes. (**D**) GO enrichment of cold-downregulated genes.

PCA results (Figure 2B) showed that the biological replicates within each treatment group clustered well. The low-temperature stress group and the control group were separated along the principal components, with no obvious outlier samples. These findings indicated that cold exposure was associated with distinct transcriptomic profiles in *S. inexpectata*.

### 3.2. Results of Differential Expression Analysis, GO Annotation, and Enrichment Analysis

A total of 1656 differentially expressed genes (DEGs) were identified between the cold-exposed group (8 °C) and the control group (25 °C) (Figure 2A), indicating that cold exposure induced distinct changes in gene expression in *S. inexpectata*.

GO enrichment analysis was performed to characterize the biological functions of the differentially expressed genes. Among the upregulated genes (Figure 2C), enriched terms in the “biological process” category included lipid metabolic process (GO:0016042) and glycolipid catabolic process (GO:0019377). In the “cellular component” category, enriched terms included chitin-based extracellular matrix (GO:0062129) and external encapsulating structure (GO:0030312). In the “molecular function” category, enriched terms included serine-type peptidase activity (GO:0008236), hydrolase activity acting on glycosyl bonds (GO:0008233), and structural constituent of cuticle (GO:0042302). These results suggested that genes related to metabolic processes and cuticular structure may contribute to the cold-stress response of *S. inexpectata*.

GO enrichment analysis of downregulated genes (Figure 2D) showed enrichment of terms related to DNA repair (GO:0006281) and signal transduction (GO:0007165). These results suggested that cold exposure was associated with reduced expression of genes involved in genome maintenance and cellular signaling in *S. inexpectata*.

### 3.3. Results of Comparative Transcriptomics and Domain Identification

**Figure 3:**
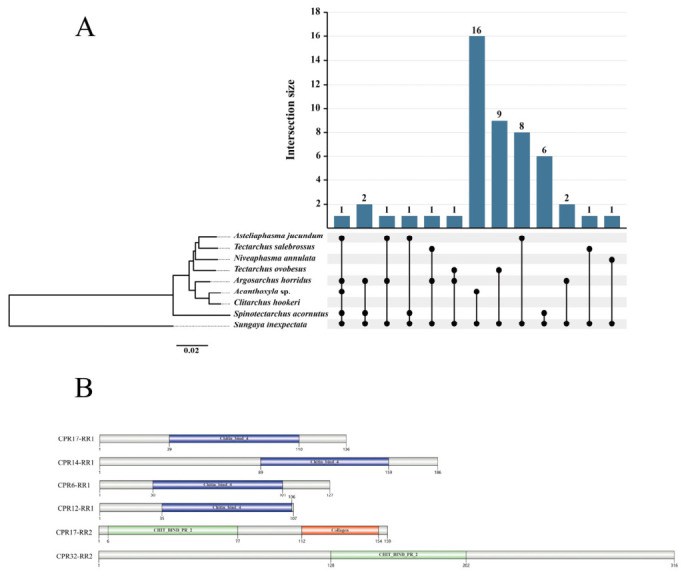
Cross-species comparison of cold-upregulated orthogroups and domain architecture of selected cuticular proteins. (**A**) Cross-species UpSet analysis of cold-upregulated orthogroups in nine stick insect species. (**B**) Domain architecture of six selected differentially expressed cuticular protein genes from *S. inexpectata*.

Comparative transcriptome analysis was performed on nine stick insect species, including *S. inexpectata.* After species-specific genes were removed, shared cold-upregulated orthogroups were visualized using an UpSet plot (Figure 3A). One gene annotated as takeout was shared among five stick insect species and was upregulated under low-temperature stress. This gene was shared by the largest number of species among the cold-responsive orthogroups, suggesting that it may represent a conserved cold-responsive candidate gene among the examined stick insect species. Additionally, orthogroups present in three or more species were subjected to BLASTv2.14.0 searches. The identified proteins included chitin-binding type-2 domain-containing proteins, C-type lectin domain-containing proteins, beta-hexosaminidase, cuticular proteins, and lipase domain-containing proteins. Detailed parameters for the other six orthogroups that were significantly upregulated in three or more species are provided in Table S3.

Domain analysis of differentially expressed genes encoding cuticular proteins from *S. inexpectata* showed that two chitin-binding domains (Figure 3B), Chitin_bind_4 (PF00379) and CHIT_BIND_PR_2, were present in most of these genes. However, the positions of these domains within the protein sequences varied considerably. This variation may reflect structural differences among different cuticular proteins. A small number of genes contained a collagen domain.

**Figure 4:**
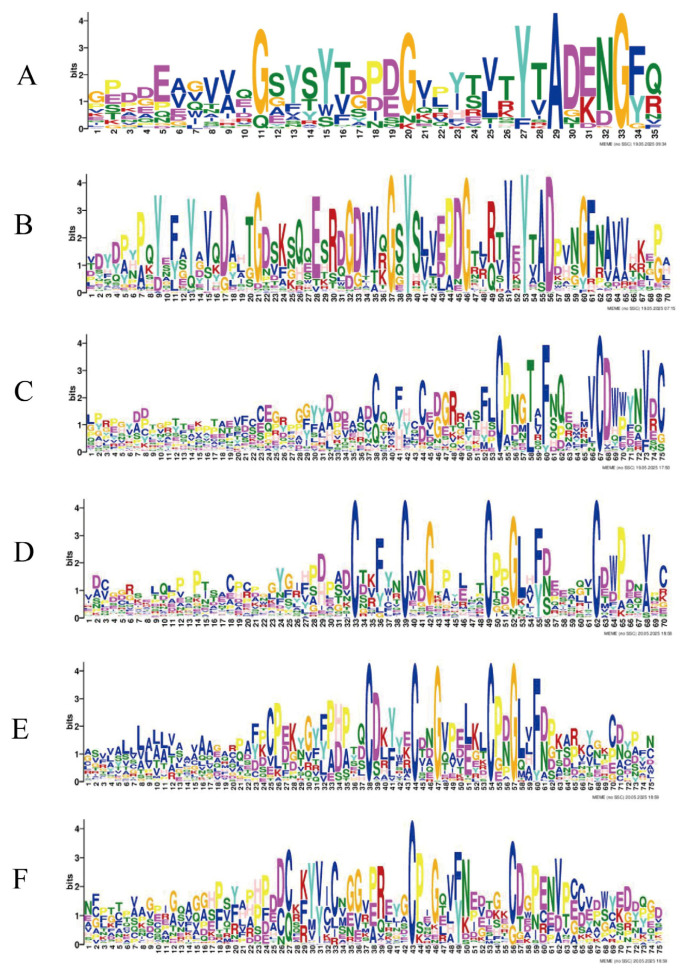
Motif structures of CPR-RR1, CPR-RR2, CPAP1, and CPAP3 cuticular proteins from nine stick insect species. (**A**) Motif structure of CPR-RR1 cuticular proteins. (**B**) Motif structure of CPR-RR2 cuticular proteins. (**C**) Motif structure of CPAP1 cuticular proteins. (**D**–**F**) Motif structures of CPAP3 cuticular proteins.

We analyzed the domain architectures of cuticular protein families from nine stick insect species, including *S. inexpectata* (Figure 4). The conserved RR1 and RR2 sequences were similar to those reported in other insects, such as *Antheraea pernyi*. For the RR1 family, the predicted RR1 domain was often incomplete. Most sequences lacked several C-terminal amino acids compared to the canonical RR1 model. Only a minority of sequences contained a full-length RR1 domain, whereas most appeared truncated. This pattern may reflect technical limitations or genuine biological variation. For the CPAP family, clear motifs with five consecutive cysteines (C) were observed. The CPAP3 family showed a motif pattern of three repeats: _13–24_ Cx_5_Cx_9–10_Cx_12–16_Cx_7–8_C. The CPAP1 family showed a single motif of _14–16_ Cx_5_Cx_9–13_Cx_12_Cx_7–8_C, which was highly consistent with the predicted pattern and supports the reliability of the CPAP1 annotation. Overall, the domain architectures of the major cuticular protein families were broadly consistent with those reported in other insect species.

### 3.4. Phylogeny of the Cuticular Protein Gene Family and Selection Pressure Analysis

**Figure 5:**
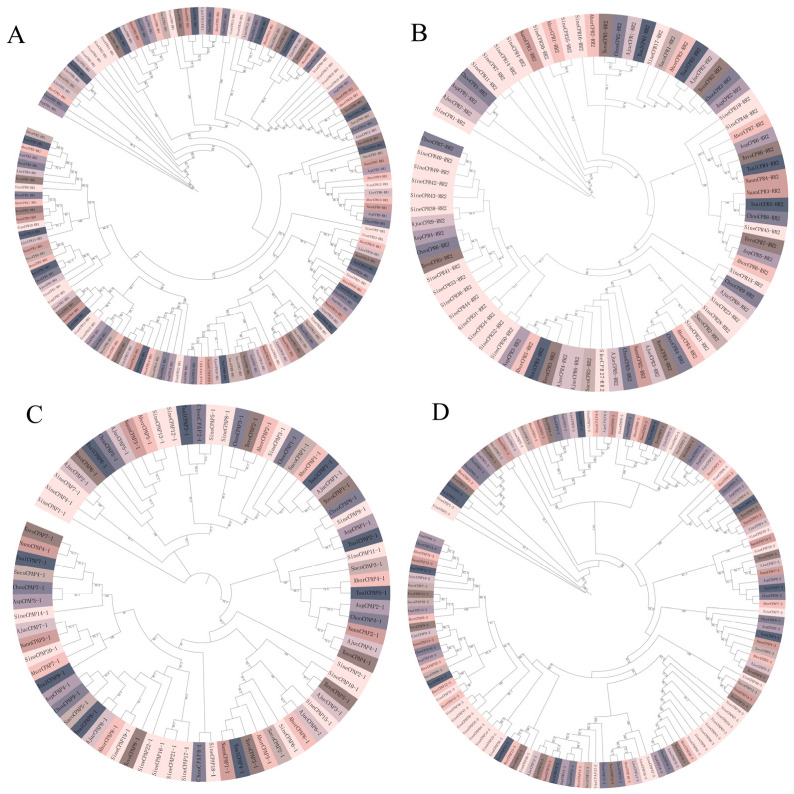
Maximum-likelihood phylogenetic relationships of CPR-RR1, CPR-RR2, CPAP1, and CPAP3 cuticular proteins from nine stick insect species. Sequence names were abbreviated using a prefix consisting of the genus initial and the first three letters of the species name, for example, *S. inexpectata* → Sine. Full names were generated by combining the prefix with the original gene name, for example, *SineCPR17-RR2*. (**A**) CPR-RR1 proteins. (**B**) CPR-RR2 proteins. (**C**) CPAP1 proteins. (**D**) CPAP3 proteins.

Phylogenetic trees of cuticular protein subfamilies were constructed using sequences from nine stick insect species. Comparisons within each cuticular protein subfamily showed that several *S. inexpectata* sequences occupied distinct positions in the phylogenetic trees, whereas others clustered with homologs from the other stick insect species. Based on this pattern, we identified eight *S. inexpectata* genes in CPR-RR1 (Figure 5A), six in CPR-RR2 (Figure 5B), six in CPAP1 (Figure 5C), and eight in CPAP3 (Figure 5D).

Selection pressure analyses detected a significant positive selection signal in one CPAP3 ortholog, *CPAP3-3*, with an *ω* value of 2.42 (*p* < 0.05). After Benjamini–Hochberg FDR correction, this signal remained statistically significant (*q* = 0.0175) (Table S4). To evaluate the reliability of this result, we further assessed the quality of the sequence alignments used for *CPAP1-1, CPAP3-3, RR1-4*, and *RR2-1*. Gap proportion statistics showed that most alignment columns contained low levels of missing data; for example, in *CPAP3-3*, 93.86% of columns had no gaps and 95.45% had no more than two gaps (Table S5). Column identity statistics also indicated high overall conservation, with mean identity values ranging from 0.906 to 0.971 across the four genes (Table S6). These alignment quality metrics supported the reliability of the selection pressure analysis. In contrast, *RR1-4, RR2-1*, and *CPAP1-1* showed ω values below 1, consistent with purifying selection and functional constraint (Table 2).

### 3.5. Results of qRT-PCR Expression Assessment

**Figure 6:**
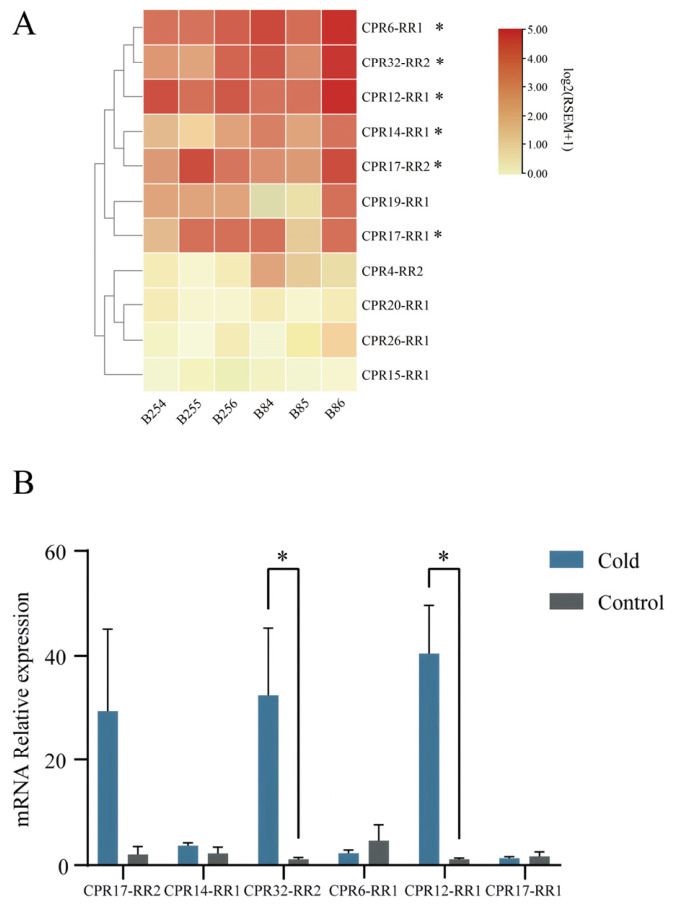
Expression profiles and qRT-PCR analysis of selected cuticular protein genes in *S. inexpectata* under low-temperature stress. (**A**) Heatmap showing the expression patterns of differentially expressed cuticular protein genes in the cold-exposed group (8 °C) and the control group (25 °C). Expression values are shown as log_2_(RSEM + 1). The prefix “Sine” was omitted from gene labels, and genes marked with asterisks were selected for qRT-PCR analysis. (**B**) qRT-PCR expression analysis of six selected cuticular protein genes in *S. inexpectata*. Bars represent relative expression levels, and error bars represent SE. Asterisks indicate statistically significant differences between the cold-exposed and control groups.

Based on the transcriptome analysis, differentially expressed cuticular protein genes in *S. inexpectata* were identified (Figure 6A). Six strongly upregulated cuticular protein genes were selected for qRT-PCR expression assessment. Among these six genes, *SineCPR32-RR2* and *SineCPR12-RR1* were significantly upregulated under cold exposure, with *p* = 0.0278 and *p* = 0.0225, respectively. Their expression levels were substantially higher than those in the control group. In addition, *SineCPR17-RR2* showed increased expression under cold stress, although the difference was not statistically significant (*p* = 0.1802). The other three genes showed no significant expression differences (Figure 6B). Although only two of the six genes showed statistically significant changes, both exhibited marked upregulation. These results suggest that *SineCPR32-RR2* and *SineCPR12-RR1* are candidate cold-responsive cuticular protein genes.

## 4. Discussion

### 4.1. Responses of Heat Shock Proteins and Cuticular Proteins to Cold Stress

To explore the functional categories associated with cold-induced transcriptional changes, we performed GO enrichment analysis of genes upregulated under low-temperature exposure in *S. inexpectata*. The enriched terms included oxidoreductase activity, hydrolase activity, proteolysis, and structural constituent of cuticle, suggesting that metabolic regulation, protein homeostasis, and cuticular structure may be involved in the cold-stress response of this species. Similar enrichment patterns have also been reported in transcriptomic studies of *Locusta migratoria tibetensis* under cold stress [53], indicating that these functional categories may represent common components of insect responses to low temperature.

Heat shock proteins (Hsps) are closely associated with stress-induced protein homeostasis. Many Hsps function as molecular chaperones, and some also possess ATPase activity, a form of hydrolase activity. In addition, Hsps can interact with proteolytic pathways to prevent protein misfolding and maintain cellular stability under stress. Previous studies on diapausing insects, such as the flesh fly *Sarcophaga crassipalpis*, have shown that many Hsp genes are upregulated during diapause, with some Hsp70 family members induced at the onset of diapause and maintained at high expression levels throughout overwintering. Consistent with these findings, we identified a cold-induced Hsp70 family member, *hsp70c*, in *S. inexpectata*. This gene was significantly upregulated under low-temperature exposure, with an approximately 2.9-fold increase (log_2_FC = 1.527, adjusted *p*-value = 0.0168), and showed high overall expression abundance (baseMean = 3730.74). These results suggest that *hsp70c* may contribute to the cold-stress response of *S. inexpectata*, consistent with the conserved role of Hsp70 proteins in insect stress tolerance.

In addition to Hsp-related responses, we identified multiple cold-upregulated genes encoding cuticular proteins, with the CPR and CPAP families representing the dominant groups. This pattern is consistent with previous studies of New Zealand stick insects, in which cuticular protein genes were associated with low-temperature responses [11,12,13]. Although the physiological mechanisms linking cuticular proteins to cold tolerance remain incompletely understood, increasing evidence suggests that the insect cuticle may play an important role in cold acclimation [11,54,55,56,57]. Changes in cuticular protein composition can alter the physicochemical properties and structural organization of the cuticle [58], which may influence desiccation resistance, barrier function, and mechanical stability under environmental stress. Similar cold-induced upregulation of cuticular protein genes has been reported in Coleoptera, Lepidoptera, and Hemiptera [12,55,59]. Our findings extend this pattern to Phasmatodea and suggest that cuticular protein genes may represent a broadly shared component of insect transcriptional responses to low-temperature stress.

### 4.2. Lipid Metabolism Under Cold Stress

GO enrichment analysis showed that several lipid-related terms were enriched under cold stress, including lipid catabolic process, membrane lipid catabolic process, and triacylglycerol lipase activity. Triacylglycerol lipase is an important enzyme involved in lipid mobilization in insects. It hydrolyzes stored triacylglycerols into free fatty acids, monoacylglycerols, diacylglycerols, and small amounts of glycerol [60]. Free fatty acids can subsequently enter oxidative metabolism to provide energy, which may support insect survival under adverse conditions such as starvation or environmental stress [61].

Triacylglycerol lipase may also be linked to membrane lipid remodeling. Previous studies have shown that this enzyme can exhibit both triacylglycerol lipase and phospholipase activities, although its affinity for triacylglycerol is higher. Its phospholipase activity may contribute to the degradation of the phospholipid monolayer surrounding lipid droplets, thereby facilitating access to stored triacylglycerols [62]. In this context, the enrichment of membrane lipid catabolic process in our dataset may reflect enhanced lipid mobilization and lipid droplet remodeling under cold exposure. In addition, glycerol produced during lipolysis can function as a cryoprotectant in some insects [63]. Therefore, the enrichment of triacylglycerol lipase activity may be associated not only with energy supply but also with the production of metabolites that contribute to cold tolerance. However, because we did not directly measure lipid composition, membrane properties, or glycerol levels, this interpretation remains preliminary. Future studies integrating lipidomics, membrane biophysical assays, and metabolite measurements, such as glycerol quantification, will be needed to test this hypothesis.

### 4.3. Selection Pressure Analysis of Cuticular Protein Genes and Potential Implications for CPAP3 Evolution

Selection pressure analysis of cuticular protein genes identified a significant positive selection signal in one CPAP3 ortholog, *CPAP3-3*. After Benjamini–Hochberg FDR correction, this signal remained statistically significant (*q* = 0.0175; Table S4). However, *CPAP3-3* did not show significant differential expression at the transcriptomic level. This suggests that the detected selection signal may be related to changes in protein sequence, structure, or biochemical properties rather than transcriptional regulation. Therefore, *CPAP3-3* represents a candidate gene for further investigation of cuticle-related adaptation in *S. inexpectata*.

CPAP3 belongs to the cuticular protein analogous to peritrophins (CPAP) family and is characterized by three type-2 chitin-binding domains (ChtBD2), each containing six conserved cysteine residues [20]. CPAP proteins are generally conserved in insects and are often expressed in cuticle-forming tissues. Previous studies have shown that CPAP proteins can contribute to cuticle integrity, molting, egg hatching, survival, and environmental adaptability [64]. In the present study, both CPAP1 and CPAP3 families were identified across the nine examined stick insect species. Functional studies suggest that CPAP3 proteins mainly affect the higher-order organization of the cuticle, including the arrangement of chitin microfibrils, their assembly into macrofibrils and laminae, and the helicoidal stacking of laminae, rather than directly altering total chitin content [65]. Thus, CPAP3 proteins are more likely to function as structural organizers of the cuticle than as enzymes directly involved in chitin synthesis. Some studies have further suggested that CPAP proteins may have functions similar to Knk proteins, such as protecting chitin from degradation and maintaining laminar organization [65], although this hypothesis requires further experimental testing.

The positive selection signal detected in *CPAP3-3* of *S. inexpectata* differs from the general expectation that structurally important CPAP3 genes are often conserved and subject to purifying selection. For example, RNA interference studies in the red flour beetle *Tribolium castaneum* showed that several CPAP3 family members, including CPAP3-A1, CPAP3-B, CPAP3-C, and CPAP3-D1/D2, are important for cuticle integrity, molting, locomotion, and survival [20]. Many of these CPAP3 genes are expressed broadly during development and are conserved across insect orders, suggesting that they perform fundamental roles in cuticle organization.

Several factors may explain why *CPAP3-3* in *S. inexpectata* showed a different evolutionary signal. First, the biological contexts differ. The *T. castaneum* study focused on normal developmental processes, including molting, metamorphosis, and adult cuticle formation, whereas our study examined cold-stress responses in a tropical stick insect. Positive selection on *CPAP3-3* may therefore reflect sequence divergence associated with environmental stress responses, particularly changes in cuticular properties under low-temperature conditions. Second, the ecological backgrounds of the two species differ. *T. castaneum* is a cosmopolitan stored-product beetle with broad environmental tolerance, whereas *S. inexpectata* is native to the tropical Philippines and may experience different selective pressures when exposed to cooler environments. Third, differences in CPAP3 gene family composition may also influence evolutionary patterns. In species with multiple CPAP3 paralogs, functional partitioning or redundancy among paralogs may lead to different levels of evolutionary constraint across individual genes.

Despite these differences, both our results and previous functional studies support the importance of CPAP3 proteins in cuticle organization. In *T. castaneum*, disruption of CPAP3 function causes cuticular defects such as wrinkled elytra, dimpled pronotum, and joint abnormalities, without necessarily changing total chitin content [20]. In our study, we did not perform functional knockdown or biochemical assays; therefore, the role of *CPAP3-3* in cold adaptation remains hypothetical. Nevertheless, the positive selection signal detected in *CPAP3-3* suggests that this gene may have undergone sequence-level divergence potentially related to cuticular structural adaptation. Future studies combining functional assays, structural modeling, and experimental cold-tolerance tests will be needed to determine whether *CPAP3-3* contributes directly to cold adaptation in *S. inexpectata*.

### 4.4. Limitations of the Study

We have identified several limitations of this study that should be acknowledged. First, the experimental design used a single acute cold exposure (8 ° C for 24 h) and whole-body tissue homogenates, which captures only an early transcriptional snapshot. Consequently, temporal dynamics and tissue-specific responses were not addressed. Second, our positive selection analysis did not include an outgroup, which may affect the accuracy of foreground branch assignment. Although the phylogenetic topology was consistent with an outgroup-validated tree from a parallel study, the lack of an outgroup in the present phylogenetic tree remains a technical constraint. Third, orthology inference relied on de novo transcriptome assemblies of nine stick insect species. Although we retained only the longest transcript per locus and applied stringent filtering, some degree of assembly-driven bias cannot be completely excluded. Future studies should overcome these limitations by using multiple time points, dissected tissues, and improved transcriptome assembly strategies, as well as including proper outgroups for selection analyses.

## 5. Conclusions

In this study, we characterized the transcriptomic response of the tropical stick insect *Sungaya inexpectata* to acute cold stress (8 °C), identifying a large set of differentially expressed genes, with cuticular protein genes from the CPR and CPAP families prominently upregulated. Selection pressure analysis indicated that most cuticular protein genes are under purifying selection, whereas *CPAP3-3* showed evidence of positive selection (*ω* = 2.42, *q* = 0.0175), suggesting potential structural or biochemical modifications of this protein under environmental pressure. These findings highlight the potential importance of cuticular proteins in cold-stress responses and provide a set of candidate genes for future functional studies. The transcriptomic dataset generated here offers a valuable resource for understanding cold-stress responses in tropical ectotherms. Future work will include comparative analyses across a broader range of stick insect species to explore the relative contributions of developmental constraints and environmental factors to the evolution of CPAP3 genes.

## Figures and Tables

**Table 1:** Quantitative Primer Sequences of Cuticular Protein Genes.

Gene	Forward Primer (5′ to 3′)	Reverse Primer (5′ to 3′)	Amplicon Size (bp)
*β-actin*	CGCCATGTATGTCGCCATCCAG	AAGCTGTAGCCACGCTCTGTCA	209
*Sine*CPR17-RR2	ACACCAAGTCCCAGCAGGAGAC	AGGTTTGACGGCGACCACTG	211
*Sine*CPR14-RR1	CGAACGCAGTCTCCAAGGAACC	TGGACCTAAGGATGGCCTCTGG	170
*Sine*CPR32-RR2	AGCCGACTAGCATCGTGGTGAA	CGAACTGCTGAGGCGTCTGTTG	221
*Sine*CPR6-RR1	GGCACCAAGGTTGACGCATCA	GCCAGAGCCTCCAGGATCTTCT	205
*Sine*CPR12-RR1	TGAAGGTGGTGCTCGTCTGTCT	TTGATGCCGTTCGCCGTCTC	150
*Sine*CPR17-RR1	AACAGTTACGAGAGCGGTGACG	GCCAGAGCCTCCAGGATCTTCT	226

**Table 2:** Selection pressure analysis results of four cuticular protein genes shared by all nine stick insect species.

Gene	Models Compared	2ΔlnL	LRT *p*-Value	*ω*
*CPR-RR1-4*	M2a_rel vs. CmC	4.38113	0.036338899 *	0.15286
*CPR-RR2-1*	M2a_rel vs. CmC	4.00055	0.045485420 *	0.80129
*CPAP1-1*	M2a_rel vs. CmC	4.023486	0.044870877 *	0.42284
*CPAP3-3*	M2a_rel vs. CmC	8.120402	0.004376985 **	2.42143

Note: LRT, likelihood ratio test. Asterisks indicate statistical significance before FDR correction: * indicates *p* < 0.05, and ** indicates *p* < 0.01. *CPAP3-3* remained statistically significant after Benjamini–Hochberg false discovery rate correction (*q* = 0.0175).

## Data Availability

Data to support this study are available from the National Center for Biotechnology Information (https://www.ncbi.nlm.nih.gov) (accessed on 20 December 2024). Accession: PRJNA1433133.

## Supplementary Materials

The following supporting information can be downloaded at: https://www.mdpi.com/article/10.3390/insects17060604/s1. Table S1. Fastp Report Summary. Table S2. Summary of transcriptome assembly data. Table S3. BLAST alignment parameters. Table S4. Multiple testing correction for positive selection analyses of cuticular protein genes. Table S5. Gap proportion statistics for the CPAP1-1, CPAP3-3, RR1-4 and RR2-1 alignments. For CPAP3-3, columns containing 5 or 6 gaps were excluded from downstream analyses. Table S6. Column identity statistics for the CPAP1-1, CPAP3-3, RR1-4, and RR2-1 alignments. Figure S1. BUSCO Assessment Results.
