# Supplementary material for: Cold Tolerance and Differential Expression of Cuticular Protein Genes in Sungaya inexpectata Zompro, 1996 (Insecta: Phasmatodea)
Source: Insects. 2026 Jun 8;17(6):604. doi: 10.3390/insects17060604 (PMC13301680; doi:10.3390/insects17060604)
Supplement: Supplementary file 1 [file insects-17-00604-s001.zip › SupplementaryTable.pdf]

Table S1. Fastp Report Summary

Sample	Raw Reads	Clean Reads	Mean Length	Q20 Bases	Q30 Bases	GC Content
B251	48,506,238	48,322,966	149	99.20%	97.39%	43.34%
B252	46,398,668	46,110,248	149	98.99%	96.86%	45.20%
B253	42,194,390	42,039,714	149	99.23%	97.42%	43.72%
B254	47,810,304	47,629,672	149	99.23%	97.43%	43.97%
B255	53,911,748	53,712,288	149	99.17%	97.29%	45.05%
B256	52,616,244	52,430,750	149	99.25%	97.49%	41.85%
B81	55,063,498	54,803,522	149	99.14%	97.20%	45.29%
B83	46,995,582	46,822,568	149	99.23%	97.41%	43.96%
B84	51,618,668	51,406,070	149	99.18%	97.31%	44.54%
B85	59,584,352	59,351,608	149	99.18%	97.37%	34.12%
B86	45,597,684	45,406,802	149	99.23%	97.46%	35.68%

Table S2. Summary of transcriptome assembly data

Category	Metric	All Transcript Contigs	Longest Isoform per Gene
General Assembly Stats	Total genes	302,864	/
	Total transcripts	414,062	/
	GC content (%)	40.24	/
Contig Length (bp)	N10	11,024	8,719
	N20	7,999	5,486
	N30	6,005	3,649
	N40	4,551	2,424
	N50	3,383	1,620
	Median	513	449
	Mean	1,324.39	903.25
Assembly Output	Total assembled bases	548,377,768	273,561,277

Table S3. BLAST alignment parameters

Number	Description	Identity	Score	E-value
**1**	Chitin-binding type-2 domain-containing protein	77.4%	1043	2.5e-142
**2**	C-type lectin domain-containing protein	89%	1092	8.2e-150
**3**	Cuticle protein 16.5-like	56.1%	245	1.5e-24
**4**	Beta-hexosaminidase	87.2%	2713	0
**5**	Cuticular protein	77.3%	765	4.2e-101
**6**	Lipase domain-containing protein	47.6%	1239	4.3e-163

Table S4 Multiple testing correction for positive selection analyses of cuticular protein genes.

Gene	Raw LRT *P*-value	FDR-adjusted *q*-value
CPAP3-3	0.00438	0.0175
CPAP1	0.04487	0.0727
RR1-4	0.03634	0.0727
RR2-1	0.04549	0.0727

Table S5 Gap proportion statistics for the *CPAP1-1, CPAP3-3, RR1-4* and *RR2-1* alignments. For *CPAP3-3*, columns containing 5 or 6 gaps were excluded from downstream analyses.

Gene	Gaps per column	Gap proportion (%)	Number of columns	Percentage of total columns
CPAP1-1	0	0%	360	36.14%
	1	11.11%	376	37.75%
	2	22.22%	252	25.30%
	3	33.33%	8	0.80%
	Total	-	996	100%
CPAP3-3	0	0%	1422	93.86%
	1	11.11%	18	1.19%
	2	22.22%	6	0.40%
	5	55.56%	45	2.97%
	6	66.67%	24	1.58%
	Total	-	1515	100%
RR1-4	0	0%	411	91.95%
	1	11.11%	6	1.34%
	2	22.22%	20	4.47%
	3	33.33%	10	2.24%
	Total	-	447	100%
RR2-1	0	0%	345	76.33%
	1	11.11%	56	12.39%
	2	22.22%	51	11.28%
	Total	-	452	100%

Table S6 Column identity statistics for the *CPAP1-1, CPAP3-3, RR1-4,* and *RR2-1* alig nments.

Gene	Number of columns	Max identity	Min identity	Mean identity	Standard deviation
CPAP1	996	1.000	0.429	0.906	0.112
CPAP3-3	1515	1.000	0.444	0.971	0.070
RR1-4	447	1.000	0.444	0.954	0.103
RR2-1	452	1.000	0.286	0.926	0.131
