# Supplementary figures and images for: Cold Tolerance and Differential Expression of Cuticular Protein Genes in Sungaya inexpectata Zompro, 1996 (Insecta: Phasmatodea)
Source: Insects. 2026 Jun 8;17(6):604. doi: 10.3390/insects17060604 (PMC13301680; doi:10.3390/insects17060604)

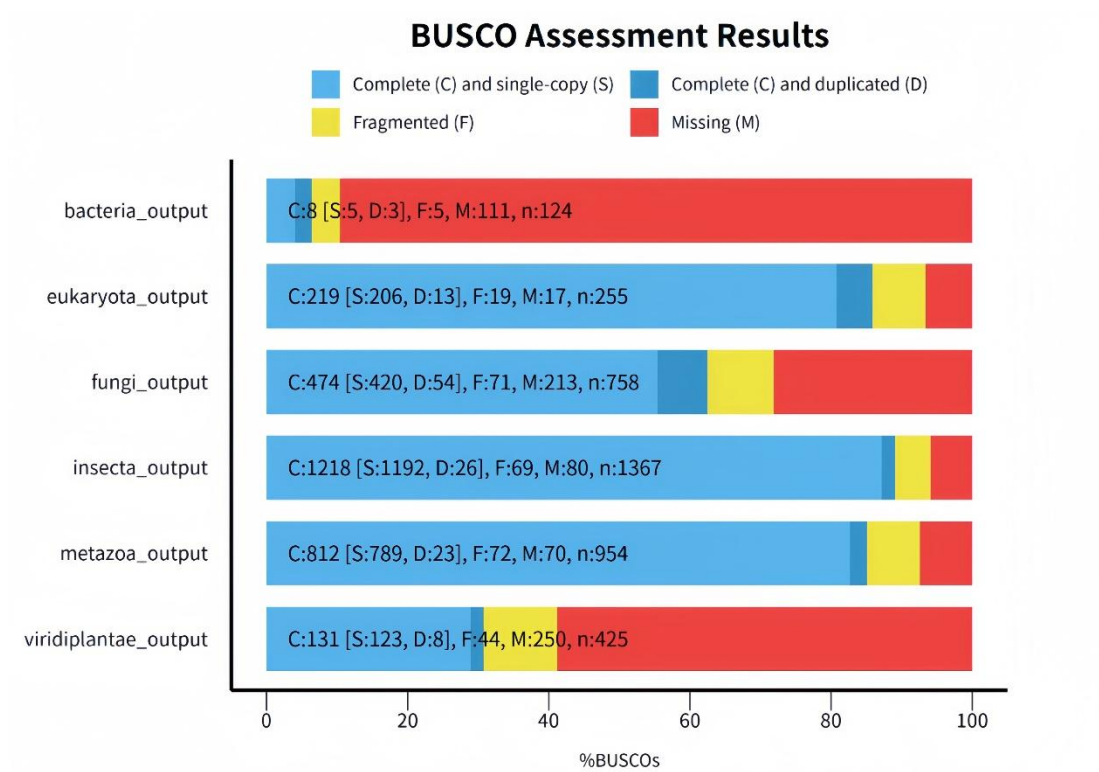

**Figure S1.** Busco Assessment Result

Supplement: Supplementary file 1 [file insects-17-00604-s001.zip › SupplementaryFigure .pdf]
